# Unveiling the Mimicker: A Case Report of Ocular Neurosyphilis

**DOI:** 10.7759/cureus.75364

**Published:** 2024-12-09

**Authors:** Daniel Ajabshir, Juan Gabriel Jimenez Garcia, Carolina Fernandez, Guillermo Izquierdo-Pretel

**Affiliations:** 1 Translational Medicine, Florida International University, Herbert Wertheim College of Medicine, Miami, USA; 2 Internal Medicine, Florida International University, Miami, USA; 3 Internal Medicine, Florida International University, Herbert Wertheim College of Medicine, Miami, USA

**Keywords:** diagnostic challenges, endophthalmitis, neurosyphilis, ocular syphilis, syphilis, treponema pallidum

## Abstract

Syphilis, an infection caused by *Treponema pallidum*, is well known for its ability to mimic other diseases across various organ systems, complicating timely diagnosis. Ocular syphilis, though rare, is a severe manifestation that can closely resemble other eye conditions, making early identification challenging. When conventional treatments fail to improve symptoms, considering syphilis in the differential diagnosis becomes crucial to avoid further complications.

We present the case of a 64-year-old woman who initially presented with progressive blurred vision, redness, and photophobia. She was initially treated for suspected endogenous endophthalmitis with broad-spectrum antibiotics, but her condition did not improve. Further diagnostic workup, including serological testing, revealed a positive result for *T. pallidum*, leading to a diagnosis of ocular neurosyphilis. Treatment with intravenous penicillin and corticosteroids resulted in a gradual improvement in her symptoms.

This case highlights the complexity of diagnosing ocular syphilis, which can mimic more common ocular infections and inflammatory conditions. A high index of suspicion is necessary when conventional treatments are ineffective, as early diagnosis and appropriate intervention are critical to preventing irreversible damage, such as permanent vision loss. Comprehensive diagnostic testing, including serological analysis, is essential to ensure accurate identification and management of this condition.

## Introduction

Syphilis, caused by the spirochete *Treponema pallidum*, is often called “the great imitator” due to its diverse clinical presentations, which can mimic symptoms of other diseases and complicate diagnosis [1]. In 2020, over 138,000 cases were reported in the United States, resulting in a case rate of 40.8 per 100,000 people, highlighting its prevalence [2]. Ocular syphilis is a rare manifestation of this infection that can occur in individuals with laboratory-confirmed syphilis at any stage of the disease, with an estimated incidence of 0.6% to 2% [2]. It can affect various structures of the eye, making diagnosis particularly challenging. The most common manifestations include posterior uveitis, panuveitis, optic neuritis, and chorioretinitis. Symptoms of ocular syphilis may include eye redness, blurred vision, and, in severe cases, permanent vision loss if not treated promptly [2-5].

Endophthalmitis is primarily caused by bacterial pathogens, which vary depending on the type and source of the infection. For instance, coagulase-negative staphylococci are frequently associated with post-cataract and post-injection cases, while *Bacillus cereus* and *Staphylococcus aureus* are commonly linked to post-traumatic and endogenous bacterial endophthalmitis, respectively [6]. This severe intraocular infection can arise from both exogenous and endogenous sources, leading to similar symptoms such as blurred vision, eye pain, and intraocular inflammation [7,8]. The overlapping symptoms can complicate clinical diagnosis, as demonstrated in a case where ocular syphilis was initially misdiagnosed as endogenous endophthalmitis.

## Case presentation

Initial presentation

A 64-year-old woman with controlled hypertension and a history of tobacco use presented with a two-week history of progressively worsening redness in her left eye, decreased vision, and discomfort. Upon ophthalmic examination, she was found to have a 0.7 mm hypopyon, granulomatous keratic precipitates (KPs), endothelial pigment, and corneal edema, all indicative of significant inflammation. Due to the worsening symptoms, the initial treatment with prednisolone acetate was escalated to difluprednate, which was administered four times daily.

Clinical course and diagnostic workup

**Figure 1:**
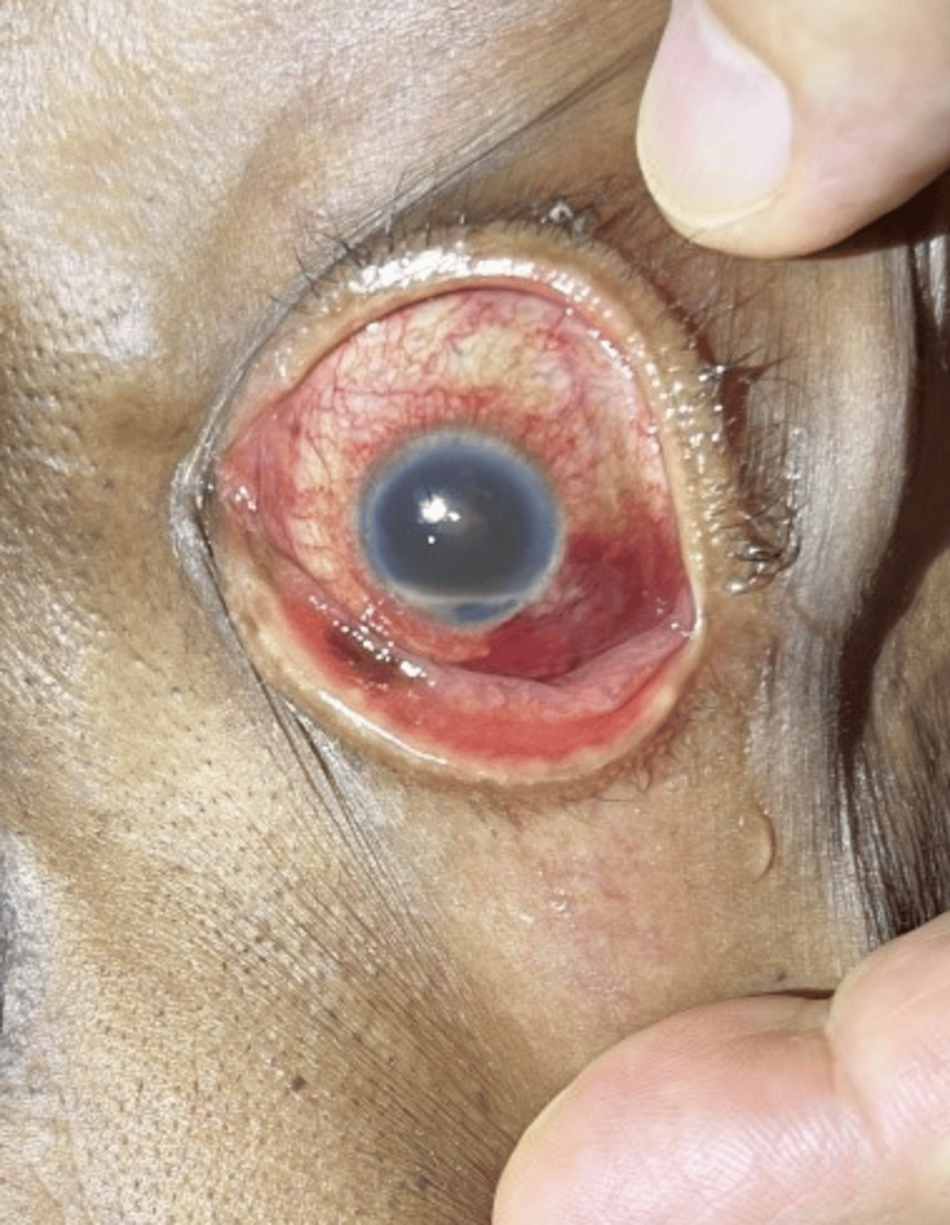
Clinical photograph showing a hypopyon in the patient’s left eye, measuring approximately 2 mm, accompanied by granulomatous keratic precipitates and corneal edema.

**Figure 2:**
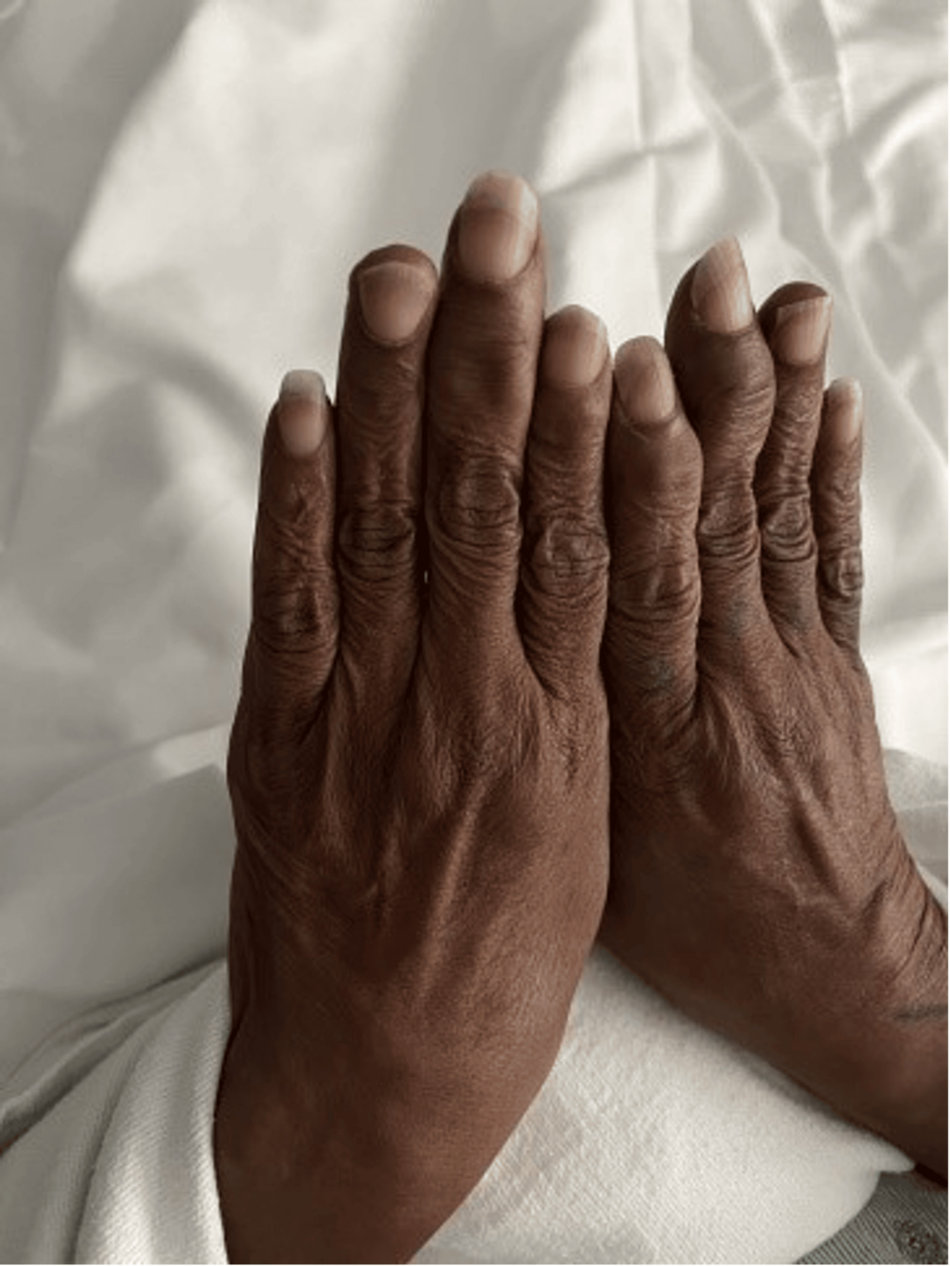
Clinical photograph highlighting the patient’s long, unkempt fingernails, which are a potential source of contamination and trauma.

Three weeks later, the patient’s symptoms had worsened. She developed new-onset headaches, decreased appetite, and further vision loss. An examination revealed a 2 mm hypopyon, worsening granulomatous KPs, and the formation of iris plaques (Figure 1). Further questioning uncovered a history of aggressive eye scratching and unkempt fingernails, raising suspicion of an infectious etiology such as endogenous endophthalmitis or *Fusarium* infection (Figure 2). Initial treatment aimed at potential bacterial infections included intravenous vancomycin, ceftazidime, and frequent use of Durezol (difluprednate) to manage inflammation. However, the patient’s symptoms persisted, prompting the need for a broader differential diagnosis.

**Figure 3:**
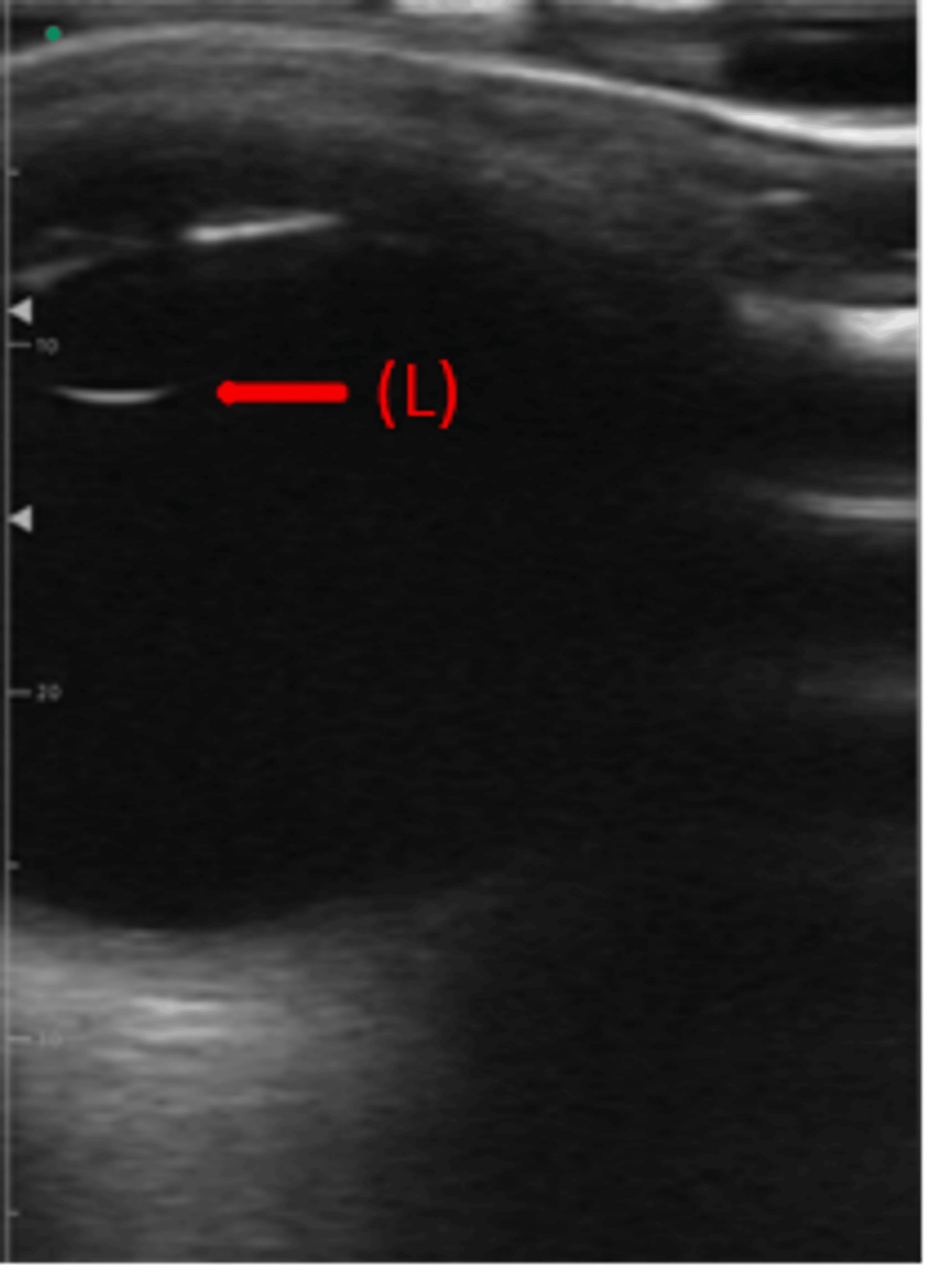
Ocular ultrasound of the patient’s left eye showing the lens (L) in a normal position without evidence of vitreous hemorrhage or structural abnormalities.

**Figure 4:**
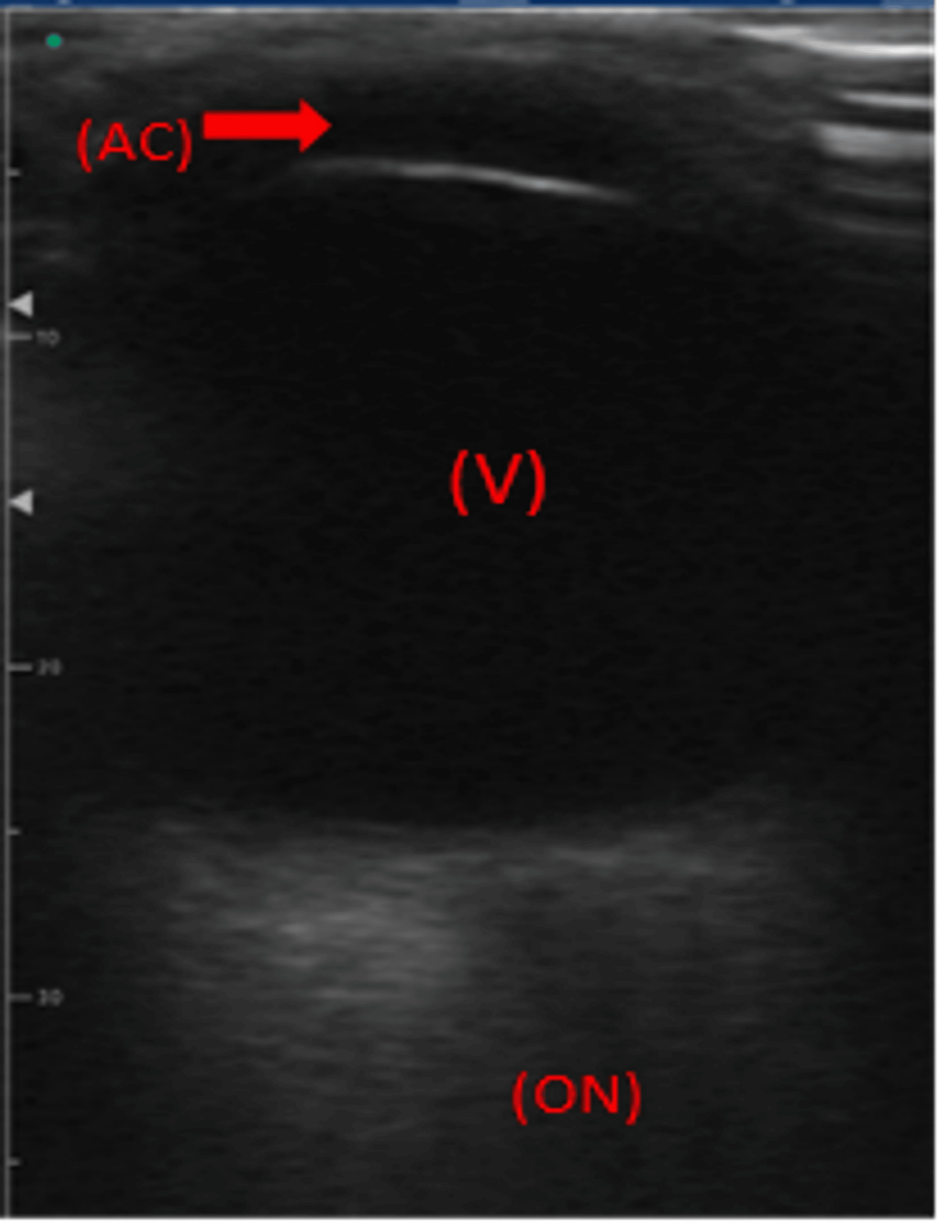
Ocular ultrasound of the patient’s left eye illustrating the anterior chamber (AC), anechoic vitreous (V), and hypoechoic optic nerve (ON) sheath in the far field, ruling out retinal detachment and increased optic nerve sheath diameter.

A bedside ocular point-of-care ultrasound (POCUS) revealed no evidence of vitreous hemorrhage, retinal detachment, or increased optic nerve sheath diameter (Figures 3-4). These findings helped rule out elevated intracranial pressure and structural abnormalities as potential causes [9]. Additional tests were conducted to investigate further systemic or localized infections, including CT imaging, echocardiography, and laboratory analyses such as rapid plasma reagin (RPR), fluorescent treponemal antibody-absorption (FTA-ABS), antinuclear antibody (ANA), and human leukocyte antigen (HLA)-B27. The results indicated the presence of antibodies to *T. pallidum* (IgG, IgM), with RPR titers of 1:256 in the serum. Cerebrospinal fluid (CSF) analysis revealed Venereal Disease Research Laboratory (VDRL) titers of 1:2 (Table 1).

**Table 1:** Comprehensive Laboratory and CSF Findings Supporting Neurosyphilis Diagnosis ACE: angiotensin-converting enzyme; Ab: antibody; AG/AB: antigen/antibody; CSF: cerebrospinal fluid; DNA: deoxyribonucleic acid; HLA-B27: human leukocyte antigen B27; IgG: immunoglobulin G; IgM: immunoglobulin M; PCR: polymerase chain reaction; RBC: red blood cell count; RPR: rapid plasma reagin; VDRL: Venereal Disease Research Laboratory; WBC: white blood cell

Test Type	Test	Result	Normal Range
Serological Tests			
	Syphilis IgG/IgM	Reactive	Negative
	RPR (Titer)	Reactive (1:256)	Negative (<1:8)
CSF Analysis			
	VDRL (Titer)	Reactive (1:2)	Negative (<1:8)
	WBC	32 cells/µL	0–5 cells/µL
	RBC	91 cells/µL	0 cells/µL
	Glucose	50 mg/dL	45–80 mg/dL
	Protein	97 mg/dL	15–60 mg/dL
	Neutrophils (%)	44%	0–6%
	Lymphocytes (%)	49%	40–80%
	Monocytes (%)	7%	2–8%
Other Tests			
	Toxoplasma Ab (IgM)	<0.2 IU/mL	Negative (<0.2 IU/mL)
	Toxoplasma IgG	<0.3 IU/mL	Negative (<1 IU/mL)
	HIV AG/AB	Negative	Negative
	ACE (Serum)	43 U/L	<40 U/L
	HLA-B27	Negative	Negative

Management

Following the diagnosis of neurosyphilis, intravenous vancomycin and ceftazidime were discontinued. The patient was then started on high-dose intravenous penicillin G at 24 million units per day for 14 days, along with corticosteroids to control inflammation. This treatment regimen led to significant improvements in her vision and resolution of ocular inflammation.

Outcome

The patient reported substantial relief from her symptoms, with no remaining signs of inflammation. Upon further questioning, she admitted to having unprotected intercourse with a single partner within the previous year, as well as a history of multiple partners, which increased her risk of syphilis exposure. Prior to this episode, she had no known history of syphilis symptoms or treatment.

## Discussion

Syphilis, caused by the spirochete *T. pallidum*, can resemble many other diseases, complicating the diagnostic process [1]. Neurosyphilis may present with neurological, psychiatric, and ophthalmological symptoms, including headaches and photophobia, making it difficult to differentiate from conditions like herpes simplex virus encephalitis, Alzheimer’s dementia, multiple sclerosis, neuroborreliosis (neural Lyme disease), paranoia, and various psychiatric disorders [10-13].

The manifestations of neurosyphilis include headaches, seizures, cognitive decline, and visual disturbances, which are also common in viral encephalitis [10,13]. For example, in one case, neurosyphilis was initially misdiagnosed as herpes simplex virus encephalitis due to MRI findings, which included persistent Fluid-Attenuated Inversion Recovery (FLAIR), signal hyperintensity in the temporal lobe that resembled those seen in herpes simplex encephalitis. The correct diagnosis was made only after laboratory tests revealed a positive VDRL and reactive RPR [10].

Ocular syphilis can develop at any stage of the disease and may affect various parts of the eye [2]. Common ocular manifestations include posterior uveitis, panuveitis, optic neuritis, and chorioretinitis, which can cause symptoms such as eye redness, blurred vision, and, in severe cases, permanent vision loss [2,5]. These symptoms overlap significantly with those of other ocular diseases, such as sarcoidosis, tuberculosis, toxoplasmosis, herpetic infections, and bacterial endophthalmitis caused by organisms like *Staphylococcus*, *Streptococcus*, *Pseudomonas*, and other Gram-negative bacteria [14,15]. Table 2 highlights key differences between endophthalmitis and ocular syphilis, emphasizing their overlapping features. In our case, the initial treatment involved broad-spectrum antibiotics for presumed non-spirochete bacterial endophthalmitis. However, the patient’s symptoms did not improve with vancomycin and ceftazidime, consistent with other reports of ocular syphilis misdiagnosed as other infections [3,7,8].

**Table 2:** Key Features Differentiating Endophthalmitis and Ocular Syphilis RPR: rapid plasma reagin; VDRL: Venereal Disease Research Laboratory

Feature	Endophthalmitis	Ocular Syphilis
Cause	Bacteria or fungi, often after surgery or trauma	Syphilis infection spreading to the eye
Symptoms	Eye pain, redness, vision loss, and pus in the eye (hypopyon)	Blurred vision, redness, and signs like uveitis
Diagnosis	Clinical examination, ocular imaging, and microbiological analysis of intraocular fluids	Blood tests for syphilis (RPR, VDRL) and spinal fluid testing
Treatment	Antibiotics or antifungals, sometimes surgery	Intravenous penicillin and sometimes steroids

Radiological and neuroimaging findings associated with neurosyphilis include hyperintensities in the mesiotemporal lobe, similar to those seen in viral encephalitis, as well as cerebral and spinal column vasculitis and optic neuritis [13,16]. Additionally, optic neuritis caused by syphilis can resemble changes seen in multiple sclerosis or other ON disorders [17].

Psychiatric manifestations of neurosyphilis, such as paranoia, schizophrenia, hallucinations, delusions, psychosis, mood disturbances, bipolar disorder, cognitive deficits, and even dementia, are well-documented, although they were absent in this patient. For instance, a 45-year-old HIV-negative male presented with psychosis characterized by delusions and erratic behavior, initially resembling a primary psychiatric condition [18]. Later, this was confirmed to be neurosyphilis through serological and cerebrospinal fluid analyses [18].

This case highlights the importance of maintaining a high index of suspicion for syphilis in patients with unexplained symptoms that do not respond to standard treatments. A comprehensive diagnostic evaluation, including cerebrospinal fluid analysis and syphilis serology, is essential for diagnosing neurosyphilis and ocular syphilis. In our patient, syphilis was confirmed only after a positive RPR and a cerebrospinal fluid VDRL test, allowing for appropriate treatment with a full-dose beta-lactam antibiotic. Early recognition and treatment are crucial for preventing complications such as permanent vision loss, cognitive decline, and other serious outcomes of neurosyphilis.

## Conclusions

Ocular syphilis, while rare, is a serious condition that can threaten vision and often resembles other eye diseases. When standard treatments do not work, it is essential to consider syphilis as part of the differential diagnosis. Early detection and prompt treatment are vital to prevent complications, including vision loss. This case highlights the necessity of thorough diagnostic evaluations, including serological testing, for the accurate identification and management of ocular syphilis.
